# Antiseptic Treatment of Alveolar Abscesses

**Published:** 1881-11

**Authors:** Wm. A. Pease

**Affiliations:** Dayton, Ohio.


THE
AMERICAN JOURNAL
OF
DENTAL SCIENCE.

Vol. XV. THIRD SERIES—NOVEMBER, 1881. No. 7.

### ARTICLE I.

### *Antiseptic Treatment of Alveolar Abscesses.*

BY WM. A. PEASE, DAYTON, OHIO.

In the June number of the *Dental Cosmos*, there is an article copied from the *British Medical Journal*, under the above title, that seems to me to be so full of errors, to be so wanting in sound pathological knowledge, that I am tempted to offer a few observations on it, even at the risk of being considered a hobbyist. There should be some principles settled in dentistry that are not to be disturbed for a light cause; that should have some of the force of an axiom, and be accepted as much as that two and two make four. Among these, is the cyst theory, i. e. the explanation of that morbid, chronic condition that occurs at the end of a root of a tooth, whose nerve has been destroyed, that is generally called an ulcer. All of the phenomena attending it, answer to the conditions of a cyst in other parts of the body with as much precision as the different conditions will admit. They make it philosophical, and it is wholly unphilosophical on any other theory. The sack is a shield that is interposed as a protection between a sensitive part and a source of irritation, that co-exists with the irritation, subject to inflammation and degeneration, as are other cysts from cause; but which, the disturbing cause being removed, will be reformed, or destructive inflammation will supervene. The parts in which these cysts occur are exceptional, both in structure and exposure to irritation, but the modifications which they entail are slight. The causes that produce it are essentially two:

1st. That which arises from the sudden stopping of a blood current, before the anastomosing vessels have had time to provide for it.

2nd. The presence of decomposing animal matter in the pulp-canal.

As the first is unavoidable, I will pass it and proceed to the consideration of the second, which falls under the title of this article, and will quote what Mr. Arthur S. Underwood, M. R. C. S. L. D. S., says pertinent to it:

"Those who have carefully followed the antiseptic theory, as practised and taught at King's College, by Prof. Lister and Mr. Cheyne, will find no difficulty in believing that, if an inflamed tract can be rendered aseptic or free from, and inaccessible to germs, that tract will heal; in fact, that it cannot help healing; moreover, if a slough be rendered aseptic, it will not be ejected from the economy by the violent methods of inflammation and suppuration; but, being no longer an irritant, nor in any sense behaving as a foreign body, it will be removed imperceptibly and gradually by absorption, and replaced as imperceptibly by new and healthy tissue. This will happen as certainly in the case of the dead contents of a pulp-cavity, as it will in the case of a slough on the leg or arm, if it can be rendered aseptic, of course, if the contents of a pulp-cavity be suppurating, and the slough, with its living mass of bacteria, be shut up intact by means of a stopping, an alveolar abscess must ensue; equally certainly, if the bacteria be destroyed and the stopping be inserted, the healing process will be accompanied by no disturbance whatever; the only difficulty is to find an agent capable of effecting the destruction of the germs."

That is pretty positive; and if it be true, it simplifies the practice of dentistry amazingly, and makes it possible to save many hitherto unsaved teeth.

He continues:—"Carbolic acid would do this, but there are two objections to its use in this situation. 1st. If used too strong, its destructive effects upon the tissues are too great. 2nd. If used diluted, its effects are too transient. Now, eucalyptus oil and iodoform are antiseptic agents of a much more powerful and permanent kind, and they cause no irritation or destruction whatever of the tissues."

Now there is evidently, confusion of thought or loose writing here. This paragraph is connected directly with the one quoted above, and it should apply to the application of carbolic acid to the dead pulp of a tooth; without removal, without other treatment or the waste of time, and the tooth may be immediately filled with safety. But the qualification; "if used too strong, its destructive effects upon the tissues are too great," would seem to apply to the sack at the end of the root, as we cannot conceive of too great destruction of dead tissue by an antiseptic agent. If applied to the dead pulp in the tooth, he says it destroys what it prevents from being destroyed—a fallacy that evidently was not intended, but, which would escape the attention of a careless reader, and lead him into error, if error there can be, where both propositions are absurd. That dead tissue in favorable conditions of the system can be absorbed, especially puriform or other exudations, there can be no doubt; but they must be in contact with the absorbing surfaces; there is yet to be found an authentic case where they have sucked such matter through a tube; and a dead pulp in a tooth, like an inverted cone, resting on a mere point in contact with live tissue, has hitherto obstinately refused to absorb. Dead pulps may be dessicated at times, and reduced to very small proportions, but the substance is there, and it will remain unless removed by artificial means. Dessication, however, is rarely met with; and it seldom occurs except in those rare, healthy constitutions that have not been contaminated by a cross of blood or hereditary taint. In the ordinary cases that come under treatment, they are juicy enough, and continue to be so until decomposition has taken place, and they cease to be active irritants. In this condition, everything ordinarily remains quiescent for an indefinite period of time, when the periosteum has escaped the usual accidents from gas, or the congestion from the sudden stoppage of blood above mentioned; unless particles of the dead pulp are forced by the pressure of food, in mastication, through the foramen; or the same result is accomplished by the dentist while endeavoring to fill to the apex.

Medical men and dentists must always have a hobby—something new to break the monotony of routine, and at present that of bacteria is being severely ridden.

That the air is full of disease or life germs, (for the result of each is the same) cannot be doubted—microscopic parasites, ever seeking for an abraded surface or a point of lowered vitality on which to live and propogate; they are hasteners and producers of putrefaction; and it is believed their mission is ended when that process is completed. If any remain after that, they are probably an unlucky brood, born to a barren inheritance, where thrift would follow emigration, or, some laggard camp follower too indolent to thrive. It is highly improbable that they penetrate through the pulp to the foramen, and through that to the periosteum, to create a disturbance there. All of the phenomena can be better explained without them than with, and it is unwise to call in an unnecessary cause, or auxillary to elucidate what is already clear. If they are the cause of periostial disturbance, their action is partial and not general, for there are in many mouths, teeth whose pulps have long been dead, some of which give disturbance and some do not. And there is just as great a diversity of action in the pulps of teeth undergoing active decomposition. Carbolic acid will not arrest putrefaction in a pulp, even though it is applied repeatedly; and when it is carried deep down to the remains, or shreds of a pulp, in a root, inaccessible to a removing instrument, the same phenomena occurs in the same order of sequence as when it is not used; but even though it does prevent putrefaction of animal matter out of the mouth, and to a certain extent in it, it only shows that there are other elements entering into the production of periostitis or a sack than it; and those are not bacteria; for they could not feed on freshly carbolized tissue. Forty years ago it was the custom to apply Arsenic acid and morphine mixed with creosote, to destroy the exposed pulp, and the next day, without removing the pulp, to fill the tooth, and the per cent. of ulceration then, was not essentially different from what it is now in the multi-rooted teeth. Then dentists would have laughed at a man who said the troubles at the root was caused by bacteria, although it was then taught that microscopic life existed in the deposits on the teeth, and contributed to their decay. Then there was but a short interval between the removal of the Arsenical paste, just sufficient to retouch the cavity, and the filling of the tooth, sufficient perhaps, for the germs to gain access to the minutely exposed part of the dead pulp, but the arsenicated tissue, perhaps containing a little morphine, and freshly bathed with creosote, must have been an uncongenial nest for bacteria. Omniverous feeders as they are, such a diet would not be conducive to a good digestion, and such a bed would be worse than the bed of Procrustes, hermetically sealed as they were, (for all dentists put in perfect fillings) with gold. It is no part of the present purpose to cast a doubt on, or to question the germ theory, or that bacteria do exist and play an important part in the life and death of man; but to ask for moderation, a little less enthusiasm, to call a halt for verification, and to know precisely where we stand. There are many difficulties in the way of accepting all that is claimed for them, that time only can determine to be real or remove. In the mean time, there should be renewed research, a diligent collection of facts and cases; arrangement and tabulation of them by different observers, so that no mistake may be made. For instance, it should be known whether carbolic acid will destroy them in all cases, and if so, of what strength, and whether eucalyptic oil is as efficaceous as it is supposed to be. It is no trifling thing to set every dentist in the land to dosing the teeth of his patients with the above named substances, or any other, unless their specific characters are established by a preponderance of probability; they should be reported as probable, but not proved. New cases out of the ordinary run will frequently occur, that will tax credibility and prove a stumbling-block to progress, that a longer experience may make easily explicable. Within a few weeks I have extracted from the jaw of two countrymen, the first superior molar, unblemished and so sound and perfect in structure, they were a delight to the eye; but there was a slight recession of the gum on the palatine side of each, and the palatine roots were dead; one of them was much discolored and without attachment, the other had a small band around it midway between the termination of the enamel and the foramen, and yet it had a sack so much distended as to give pain. The buccal roots were healthy. Now, bacteria may have followed down the root of one to the end, there caused so much irritation as to become encysted like a trichina spiralis; nay, they may have eaten their way there through the bone of the socket, but it is difficult to see how they arrived at the end of the other without destroying the integrity of that band of periosteum. But that irritation is easily explained and made comprehensible by sudden thermal changes on the unprotected tract immediately below the enamel, or by other irritants, and the gas arising from the putrefaction of that pulp, without the aid of bacteria, is amply sufficient to cause the sack. And here, I wish to place on record some conditions, germain to the above, that I have seen only during the last six or eight months, and to which I have called the attention of some others, who I thought could see and know a thing when they see it, viz: the unusual number of cases of acute periostitis that came under treatment, where the pulp was not exposed, considerable portions of comparatively healthy bone, still remain to be traversed by disease or bacteria. Ordinarily all of those cases would have been considered good for immediate plugging, without treatment or the intervention of a non-conductor, and they have materially lessened my confidence in saving exposed pulps, when we cannot place the pulp, after exposure, in as good condition as they were; and when I also found that after treatment they had as little tenacity of life as they. What was the more peculiar about them, was the often exceptionally good teeth and sound constitutions in which they occurred. After severe questioning of the patients, thermal changes did not seem to be the cause, as without promotion they were suddenly attacked with pain, the teeth as suddenly became elongated, and they were forced to seek relief. The varying and generally low barometic pressure that was accompanied by an unusual amount of rheumatism, seemed to be coincident with them; but after all, the impression remained on my mind that electricity had much to do with it. In none of these cases were there gingivic-alveolar lesions; but whenever the teeth were extracted, the periosteum was violently inflamed from neck to foramen; the whole root being red and covered with a part of the membrane, but there was no displacement or other signs of gas; and after splitting the root, the nerve, though inflamed, had not entered on the putrefactive stage. Wherever the case had been neglected until suppuration had taken place, the pus was often found immediately beneath the body of the tooth between the bifurcations, while the foramen was free from it. This was very perplexing, placing diagnosis at fault, and rendering the exploring needle of no avail. Indeed, after some failures in exploring for gas, and when topical remedies failed, I came habitually to consider that to be the cause, and extraction generally verified that conclusion.

This is an element in periosteal disease that has not hitherto attracted attention; whether it is exceptional, due to unusual climatic changes, or it is to become constant, remains to be seen. Aseptic treatment here is not indicated; it is difficult to perceive how eucalyptic oil or iodiform can reach it, even could it be diagnosed, as the distinguishing marks are as yet so ill defined as to be of little use. Iodine has been used for more than thirty years, either as a topical dressing, or confined in the pulp canal to vaporize and penetrate wherever gas can. Its effects in that location have as often been inflammatory as otherwise. It stimulated too much, and there being no absorbent glands in that vicinity, why it should be applied in a case of acute inflammation, so painful and destructive when confined between bony walls, or to a torpid cyst and cause degeneration or decay, leaving its remains and contents to irritate live tissue, is one of those questions that vexes our modern dental pathologists.

There is a growing belief that carbolic acid vapor, applied to the surface of a fresh wound, is not as beneficial as it has been thought to be. Certainly it is an impediment to healing by first intention, and the nearer large wounds can be made to heal by that process, the better. It does not prevent the necessary exudation from decomposition, and a watchful cleanliness is probably more efficient than it.

Mr. Underwood does not seem to distinguish between an alveolar abscess and a cyst. An alveolar abscess arises from the presence or onset of an irritant so sudden and violent that nature has not the time or capacity to restrain its progress; or to hedge it around with a sack. It is active, aggressive, and impetuous, carrying destruction before it, that can be measured only by the capacity or momentum of the cause. Treatment here, when diagnosed, is of no avail; it is too slow, and the best possible course to pursue is to accelerate its course and bring it to the surface before much tissue has been destroyed. It may or may not become chronic, and result in a sack. If brought quickly to the surface before the matter has had time to thicken, stagnate and become adherent to the end of the root, the prognosis is favorable, and the probabilities are that no cyst will be formed ; that the lesion will be only temporary, and of no great importance beyond the pain and inconvenience it occasions. Soon as the broken down tissue has become purulent, sufficient liquid to move, an outlet should be made for it, but not before. A lance plunged into an inflamed tract before fluctuation, while it may give a little superficial depletion, only increases the irritation, and it may give rise to a greater resolution of tissue than when nature has had her course. But a lance at the proper time is almost a specific against chronicity, especially when a drainage tube, or its equivalent, a shred of cotton wool is inserted in the cut to prevent union by first intention. These are abscesses in the ordinary sense of the term ; and they are very different from those cold, indolent and persistent sacks that are generally called ulcers. The object and intention of nature in each case is different ; in the one, to get rid of an action and temporary irritant, in the other to shield the tissue from the persistent irritation of a permanent one. Nature in the human system, in all of her moods, seeks to repair injury, and she has so adjusted the parts to each other that they second her intentions.

The root of a tooth when partially and temporarily denuded of periosteum, can be recovered, but when an abscess has been mismanaged, or neglected to that extent that its contents have coated or become adherent to the end of it, it is incapable of periosteal regeneration, and becomes a foreign substance, a constant irritant to tissue, that must be removed by ulceration or encysted.

In like manner the degeneration of these fluids or exudations, or the injections of a mischevious or meddlesome dentistry may dissolve some portions of the root and leave it rough, dead and a constant source of irritation to the surrounding tissue; unless prevented by a fluid distended sack, and acts like a soft cushion to prevent laceration or puncture from the pressure on the tooth in mastication, and renders its presence in the socket tolerable. But there are cases on the single rooted teeth where the pressure of the sack has caused so much absorption of the socket as to reach the gum, when an incision will reveal the sack, which can be easily removed and the condition of the root noted. If otherwise in good condition, and simply coated, the incrustation can be removed, when the cavity will be filled up with secondary bone, and the root covered by it without a sack. But if the root is roughened, or it cannot be thoroughly cleaned, (quite a difficult task through so small an opening on the under side of the root,) new bone will be formed as before until its progress is checked by the roughened or coated root, to which living tissue cannot attach, and a cyst will be formed as before.

Now, in all of these cases, the method of treatment to to be adopted hinges on diagnosis. If the case be recent, the probabilities are that the prevention of putrefaction in the pulp canal, whether by removal of dead tissue or by chemical means, will be suficicient; but in chronic cases the difficulties multiply, diagnosis is obscure, aseptic treatment is tedious, for the sack is constantly throwing out bioplasm to undergo a retrograde metamorphosis, and often by the time the fluctulent or curdy particles cease to appear in the root of the tooth, if they ever do, the structure of the tooth has deteriorated so much as to be of little worth.

This has been my experience, and I frequently see teeth undergoing treatment by others that any intelligent diagnosis would call hopeless, where the patience of the patient and the tooth have been treated to death. Why, if they wish to save the tooth, after the death of the cementum, they persist in destroying the crusta-petrosa is one of those unfathomable mysteries of the dental mind.

Mr. Underwood continues:—"The two points upon which I have not made myself sufficiently clear, are—1st.

The method of applying the reagents ; and, 2nd, The proportion in which I should use them.

With regard to both points, they depend entirely upon the nature of the case. Either may be used alone, or both together, in any proportion most convenient to the case in hand. Where it is necessary to inject, the oil must be used alone. In the case of alveolar abscesses, the best plan is to inject the oil every day with a hypodermic syringe, or any other syringe with a sufficiently small nozzle, the root of the tooth being dressed with wool dipped first in the oil and then in the iodoform. The iodoform will stick to the wool and subsequently dissolve it. In the case of a nerve that is partly dead, the cavity may be dressed with wool dipped first in the oil and then in the iodoform, and applied just as creosote would be, with this difference, that it is quite unnecessary to remove much of the dead tissue."

Here, again, there is a delightful uncertainty as to the meaning. He "injects the oil every day with a hypodermic syringe." Where ? Into the pulp-canal, or through a fistula or incision directly on to the sack ? The latter would seem to be the case, as he adds—"the root of the tooth being dressed with wool dipped first in the oil and then in the iodoform." A substance that would dissolve wool, would seem to be strong enough to destroy bacteria, the sack and every particle of tissue in the excavation in the socket. Instead of the re-formation of bone, necrosis would seem to be the next in order. If he meant injection through the pulp canal, how much integrity would there remain in the cementum that has been simmering in that porridge for an indefinite time ?

He closes his article thus :—"I am going to try the effect of a composition, first suggested by Mr. Waïson Cheyne, of King's College, and used by him in the form of bougies for the treatment of gonhorrhœa, composed of cocoanut butter, iodoform, and eucalyptus oil, which melts at the temperature of the body. I shall cut a small piece off of the bougie, and insert it into the pulp-cavity over the dead tissue without any wool, and cover it with a gutta-percha stopping. This proceeding, if successful, will obviate the presence of the wool, which will, of course, be a great advantage."

Now, would not the word "use," instead of "presence" be clearer and better? And after all, in the last sentence comes his first doubt.

That bacteria live in the mouth there is but little doubt. They are found in the encrustations around the necks of the teeth, they burrow beneath the gum, and feed upon, or by their irritation cause the wasting of the alveolus. It is a disease, or a condition, the cause of which has been hitherto obscure, and the names that have been applied to it have not been descriptive. Would not Bacteria Alveolaris be more significant? For a long time I have used the Tincture Iodine on the gum in such cases with favorable results. It should be allowed to run down between the gum and the teeth, freely to the border of the alveolus, when, if the encrustations have been removed, both socket and gum have become healthier than by any other means that I have used. As to the presence of bacteria on dead animal matter, or in the living organisms, much may be said. That they are the universal putrefactive or fermentative agents, is probably untrue; but much depends on climate and moisture. In the warm and moist climate of England the germs of them are, undoubtedly, abundant in the air, and a slight exposure of a decoction of meat would be sufficient to contaminate the whole.

On the contrary, in the dry air of Colorado or New Mexico, they do not seem to be abundant, if the absence of ready putrefaction has any significance, as meat hung up in the air remains uncontaminated; but a dead pulp runs through the same course of decomposition as elsewhere. Chiefly human parasites, they follow man, and wherever his filth or excreta have contaminated the soil and air, and lowered the resisting powers of his system to zymotic disease; then their invasions are the most to be dreaded. Their germs swarm in the air of old and large cities, where animal life and remains are the most abundant, and there they will the oftener be found in the mouth, but that they are the cause of what we call caries of the teeth, is so problematical that it belongs to the realm of ideality. And it is equally ideal to suppose that on the death of a pulp, they gain access to it and cause all of the phenomena that ensues to the formation of a sack, and the varying changes after it. These are more or less climatic, and they follow on the heels of thermal and barometric changes like cause or effect. But they are not alone; other abnormal and secretory phenomena follow that cannot be attributed to bacteria; that are as much dependent on them as are the livelier manifestations of the sack. Whether the low pressure of the barometer, that heralds the approach of a storm, has relieved the body of enough atmospheric weight to give to some of the nerves a greater æsthesia, or an exalted sensibility in some part of their length, or, whether their mucus or dermal terminations, taking cognition of the change, have sought to arouse and prepare the system for it, it is certain that in some constitutions, chiefly, of a more or less depraved nature, the nerves of sensation are unduly excited; while the nerves of secretion seem to be shocked, to become for the time being torpid, and the internal organs sympathize with the more exposed mucus and dermal surfaces. This action is depressing to the mind, the whole nervous system, and to all the functions of the body; and such persons are barometric as to low pressure sure and not to high, or, at least the high is attended by normal systemic action. Why the atmospheric or telluric conditions that accompany low pressure should be peculiarly favorable to parulis, why they should stir up bacteria in every old sack, if bacteria are there, is one of those question that needs serious consideration. They have not heretofore, been considered so sensitive as to arouse so easily to activity, to become voracious, or to choose that time for fission, and the letting loose of a new swarm for the production of that increased irritation, effusion, or secretion that occurs in those old sacks; and the concomitant irritations in other parts of the system where bacteria cannot exist, as in corns, the metatarsal, carpal, or other articulations, would seem to place them without the pale of reasonable consideration. At any rate they should not, at present, be made the basis of dental operations, and strong aseptics should be used sparingly. What is the most wanted is a greater care in cleansing the pulp-canal; that secured, the best results may be hoped for; but, if some portion of the pulp cannot be removed, that antiseptic that will render the remains of it the soonest inert or prevent the formation of gas, is the best. Then, wherever there is a doubt of the possibility of animal remains being in the canal after abstaining from plugging to the foramen and driving the dead matter through it, we can put our trust in Providence and feel that our duty has been fulfilled. —*Dental Register.*

